# Evaluating the Metrics of Insecticide Resistance and Efficacy: Comparison of the CDC Bottle Bioassay with Formulated and Technical-Grade Insecticide and a Sentinel Cage Field Trial †

**DOI:** 10.3390/tropicalmed10080219

**Published:** 2025-08-01

**Authors:** Deborah A. Dritz, Mario Novelo, Sarah S. Wheeler

**Affiliations:** Sacramento-Yolo Mosquito and Vector Control District, 8631 Bond Rd, Elk Grove, CA 95624, USA

**Keywords:** *Culex*, diagnostics, mosquito control, susceptibility

## Abstract

Insecticide resistance monitoring is essential for effective mosquito control. This study compared CDC Bottle Bioassays (BBAs) using technical and formulated insecticides (deltamethrin/Deltagard and malathion/Fyfanon EW) against the *Culex pipiens* complex (Fogg Rd) and *Culex tarsalis* Coquillett (Vic Fazio). BBAs indicated resistance to deltamethrin and emerging resistance to malathion in Fogg Rd, as well as resistance to both in Vic Fazio. Field trials, however, showed high efficacy: Deltagard caused 97.7% mortality in Fogg Rd and 99.4% in Vic Fazio. Fyfanon EW produced 100% mortality in Fogg Rd but only 47% in Vic Fazio. Extended BBA endpoints at 120 and 180 min aligned better with field outcomes. Deltagard achieved 100% mortality at 120 min in both populations; technical deltamethrin reached 85.7% (Fogg Rd) and 83.5% (Vic Fazio) at 180 min. Fyfanon EW and malathion showed similar performance: 100% mortality was achieved in Fogg Rd by 120 min but was lower in Vic Fazio; malathion reached 55%; and Fyfanon EW reached 58.6% by 180 min. Statistical analysis confirmed that BBAs using formulated products better reflected field performance, particularly when proprietary ingredients were involved. These findings support the use of formulated products and extended observation times in BBAs to improve operational relevance and resistance interpretation in addition to detecting levels of insecticide resistance.

## 1. Introduction

Mosquitoes are a global public health concern because female mosquitoes need to feed on blood to support their reproductive cycle, thus enabling them to vector multiple pathogens. Depending on the species, mosquitoes draw blood from a variety of hosts, including humans. Their bites can cause localized reactions, such as itching, wheals, and papules [1], and may also transmit vector-borne pathogens. Mosquitoes are competent vectors of parasites like *Plasmodium* (the causative agent of malaria) [2] and filarial nematodes that infect both dogs, *Dirofilaria immitis* (dog heartworm) [3,4,5], and humans, *Wuchereria bancrofti* (lymphatic filariasis) [6]. Additionally, mosquitoes transmit many viruses that affect human and animal health, for example, dengue [7], chikungunya [8], and West Nile [9].

Mosquito control agencies reduce interactions between mosquitoes, humans, and animals by implementing targeted interventions in both aquatic and terrestrial environments. In aquatic habitats, efforts focus on controlling immature mosquito stages, while, in terrestrial settings, adult mosquitoes are targeted. Adult mosquito populations are typically managed using insecticides to reduce their abundance and disrupt pathogen transmission cycles [10]. Chemical interventions are a central component of integrated pest management (IPM), a strategy used to control insect populations, minimize their impact on agriculture, and safeguard the health and quality of both human and animal life [11,12]. Over time, changes in environmental regulations and the development of insecticide resistance have restricted the availability of chemical tools and threatened the efficacy of remaining products [13]. Insecticide resistance continues to represent a problem for mosquito and vector control agencies by contributing to the perpetuation and emergence of mosquito and vector-borne diseases due to control intervention failures [14,15].

Mosquito control product manufacturers have responded to the growing challenge of insecticide resistance by incorporating novel, proprietary components, classified as trade secret ingredients, into the active ingredients of new product formulations. The efficacy of these adulticide products has traditionally been evaluated by vector control agencies through sentinel cage field trials, which assess the operational effectiveness of adult mosquito control applications [16]. More recently, the BBA has become a standard method for monitoring resistance in adult mosquito populations [17]. The BBA is an assay that evaluates insecticide resistance by exposing mosquitoes to a diagnostic dose of pesticides and then measuring the time it takes for the mosquitoes to succumb to exposure [18,19]. Glass bottles are coated with insecticide, and mosquitoes are observed over time for knockdown (mosquitoes are unable to right themselves in the bottle but not necessarily dead). This assay is generally performed under controlled conditions and was specifically designed to evaluate levels of insecticide resistance.

Routine resistance monitoring is essential to detect the development of resistance and to preserve a diverse arsenal of control products. This enables vector control programs to rotate insecticide classes strategically as part of an IPM approach, thereby sustaining long-term effectiveness. The effectiveness of formulated pesticides is typically measured in the field using caged sentinel mosquitoes. The caged mosquitoes are set in transects and exposed to the path of a space spray released in a manner consistent with routine mosquito control missions and according to the formulated product label. Pesticide effectiveness is evaluated by assessing the percent mortality of sentinel mosquitoes; both susceptible and field-collected mosquitoes can be tested to determine whether the spray reached the cages (all susceptible mosquitoes are dead) and whether the product is effective against field populations (field-collected mosquitoes are dead). Field trials are subject to weather and environmental factors that can alter measured pesticide effectiveness. However, data from field trials and BBA often do not align, raising persistent questions about the relationship between resistance detected through BBA and the operational performance of formulated products containing the same active ingredient.

Unlike traditional synergists such as piperonyl butoxide (PBO), which target specific resistance mechanisms but exhibit limited insecticidal activity on their own, the role and function of proprietary, undisclosed additives in modern formulations remain unclear. As public health agencies began deploying this new generation of adulticides, some important questions have emerged: could evaluating formulated products with a BBA, alongside standard field efficacy trials, provide more representative data on resistance and product performance? Furthermore, if these formulations were used in BBAs, would resistance still be detectable? And, finally, is the current time–mortality curve approach adequate for identifying both the presence and level of resistance in adult mosquitoes?

These questions underscore a critical gap in our understanding of how resistance monitoring tools relate to real-world product performance. Addressing this gap requires methodological innovation, including experimental designs that account for formulation-specific effects and more refined metrics for interpreting time-mortality data. Improved alignment between laboratory assays and field outcomes is essential for guiding mosquito control strategies and ensuring the continued utility of a shrinking toolbox of available and effective insecticides.

Previous work conducted at the Sacramento-Yolo Mosquito and Vector Control District using colonized susceptible and colonized resistant adult mosquitoes suggested a stronger correlation between field and BBA results when formulated products were used [20]. However, the question remained whether BBA results could simultaneously detect the presence of resistance and more accurately reflect the field efficacy of commercial products. To better understand the inconsistencies between BBA and sentinel cage field trials, we conducted a matched BBA and sentinel cage field trial. This included both field-collected and colony-reared susceptible *Culex tarsalis* Coquillett and *Culex pipiens* complex (Diptera: Culicidae), and we assessed two different chemical classes.

## 2. Materials and Methods

Mosquitoes: In the study area the *Culex pipiens* complex includes *Cx. pipiens pipiens* Linnaeus, *Cx. p. molestus* Forskal, and *Cx. quinquefasciatus* Say. These species are morphologically indistinguishable and can only be identified using molecular methods [21]. Molecular identification was outside of the scope of this manuscript; thus, members of this complex are referred to collectively as *Cx. pipiens*. A strain of wild-type *Cx. pipiens* was collected from Fogg Road in Sacramento County, California (38.3401, −121.4637), in August of 2023, using both CO_2_-baited and gravid traps. Gravid females were allowed to lay eggs, and a wild-type colony was established (Fogg Rd) and maintained for this study. Wild-type *Culex tarsalis* were collected from Vic Fazio Yolo Wildlife Area, Yolo County, CA (Vic Fazio: 38.5523, −121.6016) using CO_2_-baited traps. These mosquitoes were collected from the field as needed and were not maintained in colony. The two susceptible colonies used were CQ1 (*Cx. quinquefasciatus*) [22] and KNWR (*Cx. tarsalis*) [23].

Sentinel cage field trial: A trial was conducted on September, 28, 2023 in an open field in Elk Grove, CA (38.3802, −121.3412). A transect grid was established so that the space spray was evaluated in triplicate at 100 ft (30.4 m), 200 ft (61.0 m), and 300 ft (91.4 m) from the spray path. The transects were perpendicular to the spray path and oriented for the study site’s seasonally typical SW (225°) prevailing wind pattern. Fyfanon EW (FMC, Philadelphia, PA, USA) and Deltagard (Bayer, Pittsburg, PA, USA) sprays had a swath width of 300 ft (91.4 m) and were applied at mid-label rates of 0.00089 lb deltamethrin/acre (0.998 g/ha) and 0.043 lb malathion/acre (48.2 g/ha), respectively. The applications were made on the same night, once a thermal inversion had developed, using two different trucks equipped with identical ULV Foggers (London Fogger, Minneapolis, MN, USA; model # XKE), driving at 10 mph (16.1 km/h). Spray release started 500 ft (152.4 m) before and extended 500 ft (152.4 m) after the last transect line.

Two weather meters (Kestrel Instruments model #5500, Boothwyn, PA, USA) were deployed at 5 ft (1.5 m) and 30 ft (9.1 m), and droplet impingers (Leading Edge Associates, Inc., Daytona Beach, FL, USA) were set up with two 1.0 in (2.5 cm) glass, Teflon-coated microscope slides (John W. Hock Company, Gainesville, FL, USA) mounted 5 ft (1.5 m) above the ground. The impingers were turned on before the spray and turned off when the cages were collected. Slides were read within 24 h post-application using the DropVision Basic microscope and software system (Leading Edge Associates, Inc., Daytona Beach, FL, USA). The program was set to collect 200 droplets in up to 20 pictures that were used to generate a report delineating the average range and size of droplets as well as the droplet density at each sentinel cage transect location.

Female mosquitoes were sorted from each population by a brief (<1 min) anesthetization with carbon dioxide followed by sorting on a chill table. Tambourine-style sentinel cages [24] were constructed out of ridged cardboard ring that measured 5.88 in (inner diameter; 14.9 cm) × 1.75 in (height; 4.4 cm), and tulle fabric that had an approximate mesh opening of 0.04 in × 0.04 in (1 mm × 1 mm) was held on each face of the sentinel cage with a smaller outer cardboard ring that measured 6 in (inner diameter; 15.3 cm) × 0.5 in (height; 1.3 cm). Mosquitoes were added to the sentinel cages the day of the trial and then held in chest coolers in a climate-controlled laboratory with a thermostat set to 73 °F (22.8 °C) until the evening trial. Mosquitoes were provided with 10% sucrose via a cotton plug that filled the aspiration hole used to introduce mosquitoes into the cage. At each evaluation point four sentinel cages, each containing 20 female mosquitoes for each strain, were set out for each spray event [*Cx pipiens*: CQ1 (susceptible) and Fogg Rd (wild-type); *Cx. tarsalis*: KNWR (susceptible) and Vic Fazio (wild-type)]. Sentinel cages were deployed on a cage vane that self-oriented into the wind [25]. Control cages (three for each population) were deployed 1 h prior to the first spray application. The control and treatment cages spent the same amount of time in the field but were deployed at different times. For each application a new set of cages was deployed, the application was made; then, the cages were left undisturbed in the field for 30 min prior to collection. Cages were checked for mortality prior to deployment (0 h), pick up (0.5 h), and 12 h post-spray. Once collected from the cage vane and counted, the sentinel cages were placed in a 1 gal (3.79 L) zip-top plastic bag with a damp paper towel and were returned to the cooler where they were held in the laboratory until the 12 h count. Mosquitoes were considered down/dead if they were unable to right themselves or were spinning on their backs.

Bottle bioassays: The BBAs [18] were performed using both formulated products (Fyfanon EW and Deltagard) and technical-grade chemical standards (malathion and deltamethrin) on matched susceptible and wild-type populations. Fogg Rd *Cx. pipiens* were run compared to CQ1, and Vic Fazio *Cx. tarsalis* were compared to KNWR; these were the same populations used in sentinel cage field trials. Twenty-five females were placed in each bottle, and four replicates of each chemical plus four acetone-only control bottles were used per mosquito population. On several occasions a mosquito escaped during transfer to the bottle, leading to less than 25 mosquitoes per bottle. The deltamethrin chemical standard (N-11579-250 mg Chem Service Inc., West Chester, PA, USA) and Deltagard were used at a dose of 22 µg/bottle. This dose is higher than the sample diagnostic dose provided by the CDC [19]; the dose was increased so that susceptible colonies of (CQ1 and KNWR) attained 100% mortality within 1 h. The malathion chemical standard (N-12346-100 mg Chem Service Inc., West Chester, PA, USA) and Fyfanon EW were used at 400 µg/bottle dose. This dose was consistent with the sample diagnostic dose provided by the CDC [19]. Counts were performed every 5 min for the first 15 min and every 15 min thereafter for a total of 180 min. Observation periods were extended to 180 m to capture the dynamic range of mortality for more resistant populations. Mosquitoes were counted as down/dead when they were unable to right themselves or fly when the bottle was tapped.

According to CDC guidelines [19], mortality below 90% at the diagnostic time indicates resistance, mortality between 90 and 96% indicates the population is developing resistance, and mortality between 97 and 100% indicates susceptibility. For this study only technical-grade insecticides were used to make inferences about levels of resistance. Formulated products were included in the BBA primarily as a comparison to sentinel cage field trial data for assessing technical resistance or levels of resistance that resulted in control failures.

Special handling procedures were required to prepare Fyfanon EW for use in the BBA. According to the product label, Fyfanon EW “may be used undiluted or diluted with water only.” However, a key step in the BBA protocol involves diluting and applying the test compound in 1–2 mL of acetone to evenly coat the interior surface of the bottle. Initial miscibility testing revealed that directly diluting Fyfanon EW in acetone resulted in precipitation, rendering the solution unsuitable for use. To resolve this, a pre-dilution step was introduced: Fyfanon EW was first diluted at a 1:4 ratio in 200-proof ethyl alcohol (Gold Shield Chemical Co., Hayward, CA, USA), which enabled subsequent dilution in acetone without precipitation. All BBA involving Fyfanon EW were conducted using this modified coating method.

Statistical analysis: To adjust for control mortality, Abbott’s formula was used when acetone control bottles or sentinel cage control mortality was on average greater than 3% for a given strain [19,26].

To assess whether technical-grade or formulated insecticide better predicted mosquito mortality observed in the field, we compared mortality over time from two BBAs, one using technical-grade and the other using formulated product, with 12 h mortality from a field trial using the same formulated product. Percent mortality at each BBA time point (from 0 to 180 min) was compared to the mean 12 h mortality observed across replicate sites in the field trial. Root mean squared error (RMSE) was computed for each BBA product across all time points to quantify the deviation from field results. To evaluate whether one BBA more accurately matched field performance, we conducted a non-parametric bootstrap analysis with 1000 resamples to estimate a 95% confidence interval (CI) from the difference in RMSE (technical—formulated). A statistically significant difference was defined as a 95% CI that did not include zero. All statistical analyses were performed using R version 4.3.2 [27], with custom scripts utilizing the boot [28] and Metrics [29] packages.

## 3. Results

Each set of sentinel cages (control, Deltagard, and Fyfanon EW) was exposed to field conditions for one hour. The controls were out from 18:30 to 19:30 [mean temperature: 78.6 °F (25.9 °C); mean wind speed: 2.89 mph (1.29 km/h), mean wind direction: 37°], the cages sprayed with Fyfanon EW were out from 19:30 to 20:30 [mean temperature: 71.1 °F (21.7 °C); mean wind speed: 2.84 mph (1.27 km/h), mean wind direction: 233°], and the cages sprayed with Deltagard were out from 20:30 to 21:30 [mean temperature: 69.8 °F (21 °C); mean wind speed: 4.30 mph (1.92 km/h), mean wind direction: 228°]. Between the 5 ft and 30 ft weather meters, there was a 1.7 °F (0.9 °C) and 0.6 °F (0.3 °C) inversion for the Fyfanon EW and Deltagard applications, respectively. Overall, the wind direction for both applications aligned well with the 225° transect orientations. The wind had not yet changed direction at the time of control deployment.

There were no droplets collected on control slide set with the control cages. Both the Fyfanon EW and Deltagard label required spray equipment to be adjusted so that the droplet volume mean diameter (VMD) was between 8 and 30 µm (8 µm ≤ Dv0.5 ≤ 30 µm) and that 90% of the spray was contained in droplets smaller than 50 µm (Dv 0.9 < 50 µm). For all transect locations the range of droplets for the Fyfanon EW application was Dv0.5: 20.2–41.32 µm and Dv0.9: 21.6–43.16 µm. The mean droplet density was 4.8 drops/mm^2^; Deltagard was Dv0.5: 20.2–26.8 µm and Dv0.9: 29.0–41.3 µm; and the mean droplet density was 4.1 drops/mm^2^. Droplet measurements were within label specifications for both products.

**Figure 1:**
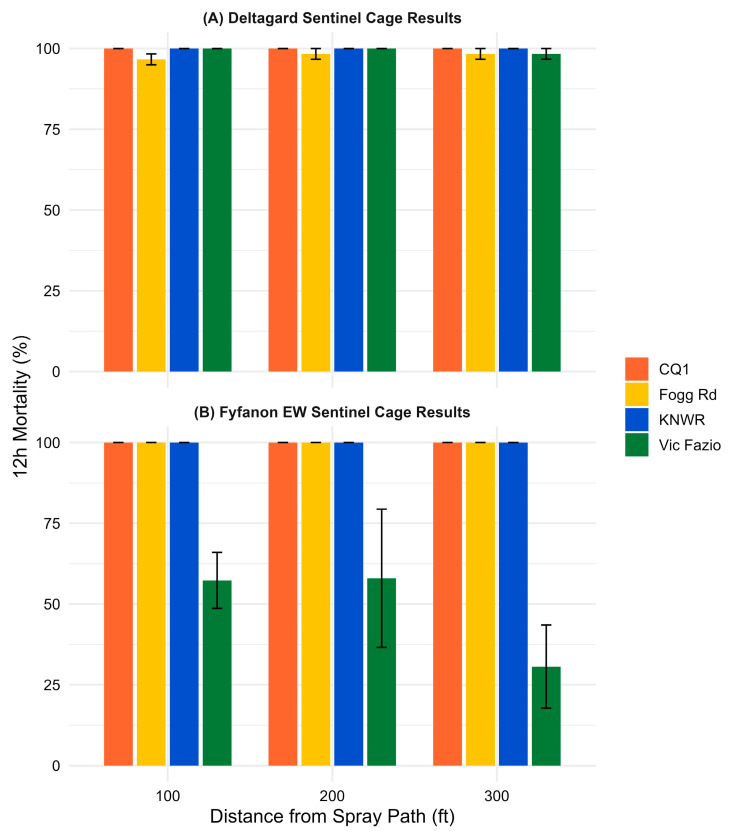
The 12 h mortality (%) in sentinel cage trials following truck-mounted applications of (**A**) Deltagard and (**B**) Fyfanon EW. Bar plots show mean ± standard error across three downwind distances: 100 ft (30.4 m), 200 ft (61.0 m), and 300 ft (91.4 m) for two strains each of *Cx. pipiens* and *Cx. tarsalis*.

All sentinel cage data are available in Table S1. Control cages had less than 3% average mortality, so Abbott’s correction was not applied. Post-spray counts at 12 h for sentinel cages exposed to Deltagard (Figure 1A) showed 100% mean mortality in both susceptible populations, CQ1 (9 sentinel cages, *n* = 164) and KNWR (9 sentinel cages, *n* = 181), indicating that the spray cloud effectively reached all sentinel cage locations. The overall mean mortality of the wild-type populations was 97.7% for Fogg Rd (*Cx. pipiens*; 9 sentinel cages, *n* = 176) and 99.4% for Vic Fazio (*Cx. tarsalis;* 9 sentinel cages, *n* = 178), suggesting that both populations were susceptible to Deltagard.

Similarly, 12 h post-spray counts for Fyfanon EW (Figure 1B) demonstrated 100% mean mortality in CQ1 (9 sentinel cages, *n* = 178), KNWR (9 sentinel cages, *n* = 180), and Fogg Rd (9 sentinel cages, *n* = 187), indicating effective product dispersion and high susceptibility in these groups. In contrast, Vic Fazio showed a substantially lower mean mortality of 47.4% (9 sentinel cages, *n* = 173), indicating reduced efficacy of Fyfanon EW in this population.

**Figure 2:**
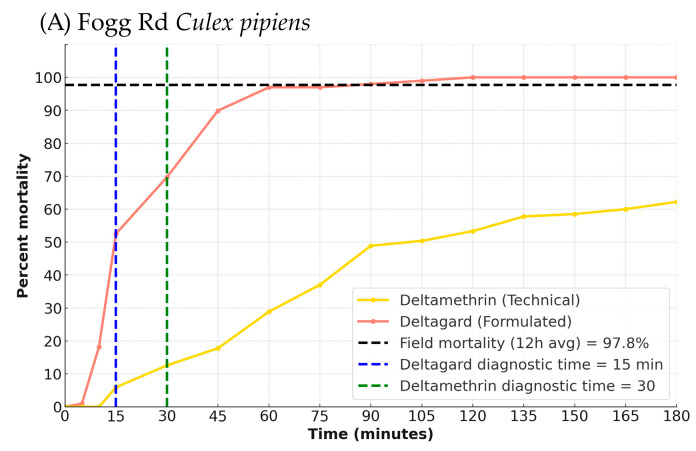

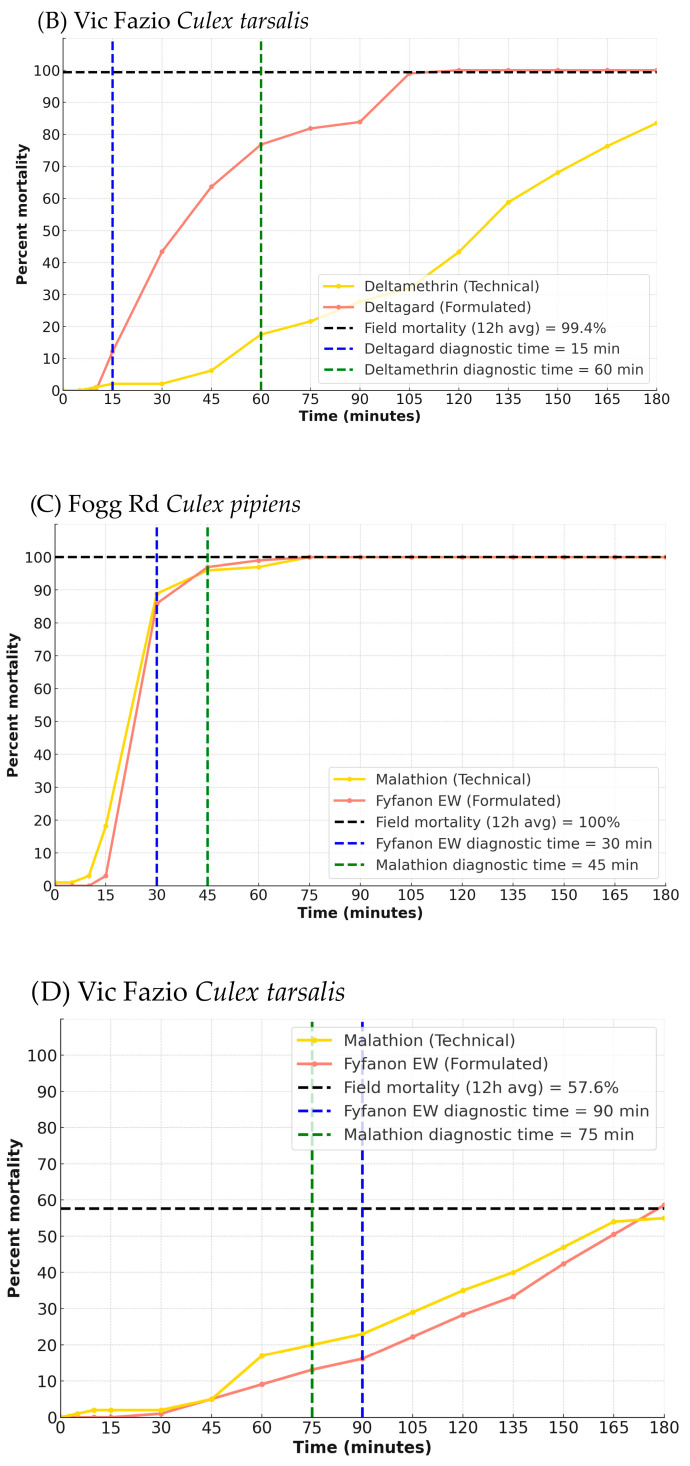
Time–mortality curves comparing formulated and technical insecticides in bottle bioassays alongside sentinel cage field trial mortality. Points represent mean mortality (±SE) over time. A dashed horizontal line (black) shows average 12 h sentinel cage mortality. Vertical dashed lines represent CDC diagnostic times for technical-grade (yellow) and formulated (orange) insecticides. (**A**) Fogg Rd *Cx. pipiens* tested against Deltagard and deltamethrin, (**B**) Vic Fazio *Cx. tarsalis* testing against Deltagard and deltamethrin, (**C**) Fogg Rd *Cx. pipiens* tested against Fyfanon EW and malathion, (**D**) Vic Fazio *Cx. tarsalis* testing against Fyfanon EW and malathion.

Across all conducted BBAs the mortality in acetone control bottles was <3%, so Abbott’s correction was not applied. All BBA data are available in Table S2. The Fogg Rd results for Deltagard and technical-grade deltamethrin are shown in Figure 2A. The diagnostic time (time at which all susceptible mosquitoes were dead or knocked down and unable to stand) was 30 min for deltamethrin (4 bottles, *n* = 99) and 15 min for Deltagard (4 bottles, *n* = 99). At the diagnostic time, mortality for Fogg Rd was 17.3% for deltamethrin and 52.5% for Deltagard (both 4 bottles, *n* = 98–99), indicating that the Fogg Rd *Cx. pipiens* were resistant to deltamethrin. However, while Deltagard-exposed mosquitoes reached 100% mortality by 120 min, deltamethrin-exposed mosquitoes only reached 85.7% by 180 min.

A similar trend was observed in *Cx. tarsalis* (Figure 2B). The diagnostic time was 60 min for deltamethrin (four bottles, *n* = 99) and 15 min for Deltagard (four bottles, *n* = 97). At these times, mortality in Vic Fazio was 17.5% for deltamethrin and 12.1% for Deltagard (four bottles each, *n* = 97–99), indicating that this population was resistant to deltamethrin. Despite low initial knockdown, Deltagard again reached 100% mortality by 120 min, while deltamethrin only achieved 83.5% by 180 min.

Bottle bioassay results for malathion and Fyfanon EW are presented in Figure 2C (Fogg Rd) and Figure 2D (Vic Fazio). In *Cx. pipiens*, the diagnostic time was 45 min for malathion (four bottles, *n* = 99) and 30 min for Fyfanon EW (four bottles, *n* = 97). At the diagnostic time, mortality in the Fogg Rd population was 96.0% for malathion and 85.9% for Fyfanon EW. The malathion data for the Fogg Rd *Cx. pipiens* indicated that the population was developing resistance. However, at both 120 and 180 min, there was 100% mortality in malathion and Fyfanon EW bottles. For *Cx. tarsalis*, the diagnostic times were 75 min for malathion and 90 min for Fyfanon EW. At these times, mortality in the Vic Fazio population was 23.0% for malathion and 13.1% for Fyfanon EW, indicating that this population was resistant to malathion. Resistance levels were also reflected in the percent mortality at 120 and 180 min. For malathion there was 35% mortality at 120 min and 55% at 180 min. Levels for Fyfanon EW were similar with 28.3% mortality at 120 min and 58.6% mortality at 180 min.

Sentinel cage and BBA analysis: For Fogg Rd *Cx. pipiens*, the RMSE between BBA and field mortality was substantially lower for the formulated product (Deltagard; RMSE = 43.35) than for the technical-grade deltamethrin (RMSE = 62.67). The bootstrapped mean difference in RMSE (technical–formulated) was 19.31 (95% CI: 8.81, 33.14). Because the confidence interval did not include zero, the difference was statistically significant, suggesting that the formulated product provided a more accurate representation of field performance. The BBA time point that most closely matched the 12 h average field mortality (97.7%) occurred at 90 min for Deltagard (97.98% mortality) and 180 min for technical-grade deltamethrin (85.7% mortality). The RMSE for the malathion BBA was 48.88, while the RMSE for the Fyfanon EW BBA was 51.39, indicating that malathion provided a slightly better match for field mortality (12 h average = 100%). A bootstrap analysis of the RMSE difference (Fyfanon EW—malathion) yielded a mean difference of 2.51 (95% CI: −2.56, 7.49). Because the confidence interval includes zero, the difference in RMSE between the two products was not statistically significant. Both products reached complete mortality at 75 min, aligning with field efficacy.

For Vic Fazio *Cx. tarsalis,* the RMSE analysis indicated that the BBA using malathion (technical grade, RMSE = 32.95) was more closely aligned with field performance (12 h average mortality = 57.6%) compared to Fyfanon EW (formulated product; RMSE = 35.61). A bootstrap comparison with a 95% CI for the RMSE difference, ranging from 101.55 to 273.13, indicated that the malathion BBA was significantly more closely matched to the 12 h average field mortality than the Fyfanon EW BBA. The closest match to field mortality occurred at 150 min for malathion (47%) and at 165 min for Fyfanon EW (50.5%). The mean adjusted 12 h field mortality for Deltagard was 99.4%. RMSE analysis showed that the Deltagard BBA (formulated) was more predictive of field performance (RMSE = 53.41) than that using technical-grade deltamethrin (RMSE = 75.79). A bootstrap analysis confirmed that this difference was statistically significant, with a 95% confidence interval of (1591.80, 4243.29). The closest match to field mortality occurred at 105 min for Deltagard (98.99%) and 180 min for deltamethrin (83.51%).

## 4. Discussion

Sentinel cage field trials and BBA are two commonly used tools to assess insecticide resistance and the operational effectiveness of adulticides against mosquito populations. The BBA was specifically designed to utilize technical-grade insecticide to enable a direct assessment of insecticide resistance levels in a controlled bioassay. However, measured resistance in a BBA does not necessarily translate to product failure in the field [30]. Additionally, there are species-level differences in insecticide susceptibility; thus, findings in one species are not universally applicable [31]. To address some of these issues, this study compared paired results from sentinel cage field trials and BBAs using susceptible and wild-type populations of *Cx. pipiens* and *Cx. tarsalis*, with BBAs conducted using both technical-grade and formulated insecticides. One key objective was to determine whether the use of formulated products in BBAs provided results that were more comparable to sentinel cage field trial outcomes. Our goal was not to replace the information provided by a BBA but instead to augment the collected data.

Our findings contribute to the growing body of work evaluating the performance of the CDC BBA and field-based efficacy outcomes. Previous work [32] has shown considerable variability in resistance differentiation using the CDC bottle bioassay, which was shown to have the lowest power, compared to topical application and the WHO tube test. However, other factors that might influence resistance detection regardless of the test can be the mosquito dry weight and relative humidity [33], underscoring the importance of methodological consistency. Additional variation in the CDC BBA can also be attributed to bottle dosing and cleaning methods [34].

Several inconsistencies were observed between the two methods. The BBA indicated resistance to technical-grade malathion and deltamethrin in both *Cx. pipiens* from Fogg Rd and *Cx. tarsalis* from Vic Fazio. However, sentinel cage trials showed near-complete mortality for these same populations when exposed to Deltagard, a formulated deltamethrin product. Although Deltagard-treated bottles exhibited delayed mortality compared to susceptible colonies, the field-collected populations still achieved 100% mortality by 120 min, whereas technical-grade deltamethrin failed to reach full mortality by 180 min. These findings suggest that the BBA using Deltagard better reflected sentinel cage outcomes than those using technical-grade deltamethrin, although mortality at the diagnostic time underestimated true product efficacy.

Comparisons between malathion and Fyfanon EW in the BBA were complicated by miscibility issues with Fyfanon EW in acetone. While pre-treatment dilution with ethanol improved solubility, it is unclear whether residual formulation inconsistencies affected bioassay results. Slightly higher diagnostic-time mortality was observed with Fyfanon EW in both field strains, but this may reflect either reduced intrinsic activity of the product or artifacts of imperfect formulation mixing. For example, diagnostic-time mortality in *Cx. tarsalis* was 13.1% for Fyfanon EW and 85.9% in *Cx. pipiens*, suggesting resistance in both populations. However, sentinel cage trials confirmed Fyfanon EW resistance only in Vic Fazio, while Fogg Rd showed full susceptibility. In this case, the malathion BBA more closely aligned with field trial results. Although the overall performance of malathion and Fyfanon EW was similar in the BBA, Fyfanon EW slightly overestimated resistance and, given the formulation challenges, offered no advantage over using technical-grade malathion. Thus, formulated products do not always provide more operationally relevant data, and further work is needed to determine where the use of formulated products is warranted.

Importantly, mortality at the diagnostic time was not a reliable indicator of field efficacy. However, mortality at later time points, particularly at 180 min, better aligned with sentinel cage field trial results. While many BBA protocols conclude observations at the diagnostic time or 120 min, our data indicated that this may lead to an underestimation of formulated product effectiveness. In our study, BBA outcomes were often comparable at 120 and 180 min for populations with delayed mortality curves that still achieved 100% mortality. For other populations, additional mortality occurred between 120 and 180 min, emphasizing the importance of extending observation time in resistance assessments.

This study analyzed a single BBA consisting of four technical replicates for each species and pesticide. Only phenotypic resistance was evaluated using the BBA. Future work is planned to evaluate more biological replicates and other important vector species to better assess the utility of formulated products and extended reading times for the BBA. There is also a need to further explore the resistance mechanisms observed in wild-type populations. For this study, modifications were made to the BBA protocol not to accommodate differences in mosquito population but instead to reflect differences between technical-grade and formulated products, especially when formulated products contained active trade secret ingredients. However, formulated products are not a replacement for technical-grade insecticides and should be used as an additive step to preserve the core insecticide resistance testing functionality of the BBA. This study demonstrated that small modifications to BBA methods can provide operationally relevant data on both the levels of resistance and the potential performance of formulated products in the field.

## 5. Conclusions

Bottle bioassays conducted with technical-grade deltamethrin showed reduced mortality compared to the BBA conducted with Deltagard and did not reflect the results observed in sentinel cage trials. According to its Safety Data Sheet (SDS), Deltagard contains 2.0% (concentration % by weight) deltamethrin and 1.35% trade secret ingredients, which likely contribute to its enhanced efficacy. In contrast, comparisons between Fyfanon EW and malathion showed minimal differences in time-mortality curves. Fyfanon EW’s SDS does not list trade secret ingredients, which may explain its comparable performance to technical-grade malathion in BBA.

These findings highlight the risk of underestimating the field performance of some formulated products when relying solely on technical-grade active ingredients in BBA. When evaluating commercial products that include ingredients that enhance the formulation, incorporating the actual product into bioassays can yield more accurate and programmatically useful resistance data. However, when the commercial product does not include undisclosed ingredients, technical-grade insecticides may provide an adequate representation of field performance, especially when the 120 or 180 min mortality is considered.

## Data Availability

All data supporting the findings of this study are included in the Supplementary Materials provided with the manuscript.

## Supplementary Materials

The following supporting information can be downloaded at https://www.mdpi.com/article/10.3390/tropicalmed10080219/s1. Table S1: Complete sentinel cage field trial dataset; Table S2: Complete Bottle bioassay dataset.

## Abbreviations

The following abbreviations are used in this manuscript:

CDC	Centers for Disease Control and Prevention
BBA	CDC bottle bioassay
IPM	Integrated pest management
ULV	Ultra-low volume
PBO	Piperonyl butoxide
Fogg Rd	A recent colony of *Culex pipiens* established from a founder population collected from Fogg Road, Sacramento County, CA
Vic Fazio	Field-collected *Culex tarsalis* from the Vic Fazio Yolo Wildlife Area, Yolo County, CA
RMSE	Root mean squared error
CI	Confidence interval
SDS	Safety data sheet
